# The Relationship between Emotion Regulation and Emotion Knowledge in Preschoolers: A Longitudinal Study

**DOI:** 10.3390/ijerph17165726

**Published:** 2020-08-07

**Authors:** Beatriz Lucas-Molina, Laura Quintanilla, Renata Sarmento-Henrique, Javier Martín Babarro, Marta Giménez-Dasí

**Affiliations:** 1 Faculty of Psychology, University of Valencia, 46010 Valencia, Spain; beatriz.lucas@uv.es; 2 Faculty of Psychology, Universidad Nacional de Educación a Distancia (UNED), 28040 Madrid, Spain; lquintanilla@psi.uned.es; 3 Department of Education and Psychology, Centro Universitario Cardenal Cisneros, 28806 Alcalá de Henares, Spain; renata.sarmento@cardenalcisneros.es; 4 Faculty of Psychology, Complutense University of Madrid, 28223 Madrid, Spain; jbabarro@psi.ucm.es

**Keywords:** emotion regulation, emotion knowledge, longitudinal study, preschool years

## Abstract

Numerous studies have shown the important role of both emotion regulation (ER) and emotion knowledge (EK) in child development. Despite the number of studies carried out on both variables, there is practically no research on the developmental relationship between these two constructs. We present a longitudinal study to explore the relationship between EK and ER in preschoolers in which we measured these variables over 3 academic years in a cohort of 108 preschool children using the Test of Emotion Comprehension (TEC) and the Emotion Regulation Checklist (ERC). The ERC is divided into 2 subscales: Emotional Regulation (ER) and Lability/Negativity (L/N). Two cross-lagged models were constructed in order to examine the predictive power of ER and L/N on EK across the three time points. The results suggest that ER is an ability that precedes and predicts EK during preschool years. We also discuss the theoretical and practical implications of these findings.

## 1. Introduction

Emotion knowledge (EK) and emotion regulation (ER) are two interrelated skills that are part of socio-emotional competence. EK can be defined as the ability to identify and understand one’s own and others’ emotional states [1]. ER is the ability to adaptively control and manage emotional states, so that emotions facilitate adaptation to situations [2,3]. Both play an important role in people’s lives and influence such important variables as psychological adjustment, health and life satisfaction [4,5,6].

Numerous studies have shown the important role that these abilities also play in the lives of children [1,7,8,9]. For example, children who show a higher EK also exhibit more prosocial behaviour, higher levels of empathy and greater acceptance by their peers [10,11,12]. Similarly, emotion regulation correlates with greater social adjustment, higher academic performance and even higher IQ scores [13,14,15,16]. In recent years, some authors have even suggested that ER is a process linked to mental health. Berking and Wuppernam [17], for example, point out that many people with mental health problems present ER deficits. In adults, disorders such as depression, anxiety, borderline personality, addictions or eating behaviour are associated with a significant deficit in ER. In children and adolescents, ER problems are a predictor of internalizing and externalizing problems [15,18,19,20]. Perhaps the most striking finding reported in the latest studies is the predictive power of ER. In their recent meta-analysis, Robson et al. [15] conclude that ER in the preschool and school years is a predictor of mental health problems, such as anxiety, depression, suicidal thoughts and aggressive behaviour 30 years later.

Given the importance of these variables, many studies have explored their pattern of acquisition in childhood and the variables involved in learning these skills. EK is acquired through different hierarchical components that become increasingly complex over time [21]. Between 2 and 3 years of age, children identify the facial expressions associated with basic emotions, and between 3 and 4 they can understand the cause of these emotions. The cultural context and the specific relevance of emotions in the family context determine whether some emotions will be better understood than others [22,23,24]. The two variables that best explain individual differences in childhood EK are the quality of attachments and linguistic interaction [25,26]. Altogether, children with secure attachments tend to exhibit better EK, and children whose parents talk more about emotions, name their emotions and explain their causes also tend to have better EK.

ER is a skill that children acquire through their relationships with adults. However, this acquisition is modulated by temperament. Some authors claim that childhood emotional problems are related to the child’s temperament and interaction with family variables [27,28]. Children with calmer and less reactive temperaments tend to have higher ER skills. Children that are high in negative reactivity have a tendency to experience negative emotions more intensely and need higher ER skills. Among the most important family variables are parental models (i.e., the ER that parents use and children observe), parental reactions toward their child’s emotions (i.e., positive vs. dismissive, punitive or negative reaction) and family climate (i.e., positive and negative emotions expressed among family members, quality of the relationship, attachment and parental responsiveness) [28].

Despite the number of studies carried out on EK and ER, there is practically no research on the developmental relationship between these two constructs. Some earlier studies have found that EK is both a predictor of and a prerequisite for ER [3,29]. These authors conclude that ER is the ability to use EK or the behavioural consequence of its use. In other words, children first recognize the emotion and understand its cause, and then adjust their behaviour accordingly. Other studies, however, have found that EK is predicted by attention and behaviour regulation skills [30,31]. Regulatory skills have been divided by some authors into so-called “hot” and “cool” executive functions [32]. The cool ones include attention control, inhibitory control and working memory. The hot ones are those specifically related to the control of emotions. Cool executive functions would be predictors of EK. The ability to control attention and behaviour are essential for focussing on emotions, identifying expressions, understanding their causes, etc. Denham et al. [33] found that although attentional and behavioural control is predictive of EK, these two variables must in fact be reciprocal (i.e., EK facilitates self-regulation and this, in turn, helps in the acquisition of EK). Although the relationship between these two skills is unclear, earlier studies ignore ER or hot executive functions in which emotion control is involved.

Our objective is to explore the relationship between EK and ER in preschoolers by means of a longitudinal study evaluating both variables at three different times points measured over a period of three years. Based on previous studies that found attentional and behavioural control to predict EK [30,31,33], we also expect ER to predict EK.

This result would highlight the importance of self-regulation skills in child development from the early years of schooling, an important factor to take into account in the design of interventional programmes. As some current theoretical models suggest, it is essential to determine the time windows in which children acquire certain skills in order to correctly time the intervention and optimize the results [34,35,36]. The learning timetable and cascade effects are part of the theoretical models that best explain development. According to these models, sensitive periods are the temporary moments that make learning or skill acquisition especially possible. These moments are key to development. In addition, the specific skills acquired will have a significant impact on later development because they impact, as a cascade, configuring the specific development path that characterizes each human being. The main contribution of this study is, from a theoretical point of view, to improve our understanding of the relationships between these two variables, and from a practical point of view, to integrate this knowledge in the design of developmental interventions.

## 2. Materials and Methods

### 2.1. Participants

This longitudinal study lasted three academic years. Participants were 108 children (51 girls and 56 boys) in total. The sample was *n* = 97 (46 girls and 51 boys) in the first wave (T1), *n* = 105 (50 girls and 55 boys) in the second (T2) and *n* = 108 in the third (T3), with a mean age of 45.71 months (range 40–51), 58.58 months (range 53–64) and 70.53 months (range 65–77), respectively. The sample number did not undergo major alterations over time. Variations in sample size were due to missing data (i.e., children who could not complete the tasks for different reasons, such as linguistic difficulties, teachers who do not return the completed questionnaires, etc.). The time elapsed between each wave was approximately 12 months (T1 = May 2014; T2 = May 2015; T3 = May 2016). Most of the children were of Spanish origin, except two Germans and one Ecuadorian. They attended a nursery school in northern Madrid (Spain). One of the authors contacted the school and presented the aims of the study to the principal and the families. The populations attending this school belong to the middle or upper middle social class. Children with atypical development were not included in the sample.

### 2.2. Measures

Test of Emotion Comprehension [37]. The Test of Emotion Comprehension (TEC) was chosen because, as stated by Pons et al. [26], it uses simple vocabulary to measure emotion knowledge. This reduces the effect of language ability on the understanding of emotions. Furthermore, it has previously been used in the Spanish population [12]. The TEC assesses emotion knowledge in children from 3 to 11 years old. The child is shown a series of cartoon scenarios and is asked to identify how the protagonist feels in each cartoon. The TEC is divided into nine components: (1) identification of basic emotions, (2) understanding of the situational causes of emotions, (3) understanding that desires can cause emotions, (4) understanding the role of beliefs in emotions, (5) understanding the role of memories in emotions, (6) understanding that emotions can be hidden, (7) knowledge about emotion regulation strategies, (8) understanding mixed emotions and (9) understanding the role of morality in emotions. A task example of understanding that desires can cause emotions is: “These two boys (girls) are very thirsty. The first boy (girl) loves Coca Cola whereas the second boy (girl) hates Coca Cola. There is a bottle of Coca Cola in the box. How is the first boy (girl) feeling when he (she) sees that there is a bottle of Coca Cola in the box? Is he (she) feeling sad, happy or nothing? How is the second boy (girl) feeling when he (she) sees that there is a bottle of Coca Cola in the box? Is he (she) feeling sad, happy or nothing?”.

For the purpose of the study and due to the participants’ age, we only considered the first six components as the last three components did not provide any discriminative information given the age of the sample. Children are expected to develop those three last components at age 8–9. TEC has shown a high test-retest correlation over 13-month period (α = 0.68) [38]. For the present study, TEC test-retest reliability was slightly lower (α = 0.65).

Emotion Regulation Checklist [39]. The ERC is a 24-item other-report measure that can be completed by parents and/or teachers. In this study the informants were teachers. Raters use a 4-point scale to indicate how often a child displays affective behaviours (1 = never, 2 = sometimes, 3 = often, 4 = almost always). The ERC consists of two subscales: Emotion regulation (ER) and emotion lability/negativity (L/N). The ER subscale captures key processes in adaptive regulation, including socially appropriate emotional displays and empathy. This subscale contains eight items, such as “is a cheerful child” or “responds positively to neutral or friendly overtures by peers”. Six items are rated positively (1, 3, 7, 14, 21 and 23), and two are inversely rated (16 and 18). We obtained each child’s score by adding the scores of all of the items, making allowances for the items that required reverse scoring. High scores indicate a greater capacity for ER. The maximum score in this subscale is 32, and the minimum is 8. The L/N subscale assesses mood lability, lack of flexibility, dysregulated negative affect and inappropriate affective displays through 15 items, such as “exhibits wide mood swings”. Eleven items are scored positively (2, 6, 8, 10, 13, 14, 17, 19, 20, 22 and 24) and four are reversely scored (4, 5, 9 and 11). The maximum score is 60, while the minimum is 15. Higher scores indicate greater emotion dysregulation. Item 12 does not score for either of the scales as it is not loaded on either factor, following ERC authors’ instructions. The ERC has shown a high reliability of α = 0.85 [40]. For this sample, internal consistency was α = 0.70 for the ER subscale and α = 0.84 for the L/N subscale.

### 2.3. Procedure

This research was approved by the Bioethical Research Committee of the National Distance Education University (UNED, Spain). This study is part of a larger longitudinal study on socioemotional development during preschool years. The principal of the participating school was informed of the purpose of the study by the research team. Parents signed the appropriate consent forms. Teachers completed questionnaires after receiving information from the research team. They had 10–15 days to return the completed questionnaire. Both the university and the school followed the protocols for applying the ethical procedures that regulate research in Spain.

### 2.4. Data Analysis

First, descriptive statistics and bivariate correlations between study variables were computed. Wilcoxon matched pairs tests were also conducted to examine mean differences across time. The correlation coefficient index (*r*) was used as the effect size estimate [40]. Second, we constructed two cross-lagged models in order to examine the predictive power of ER and L/N on EK at the three time points, and established a causal relationship between the two pairs of constructs. In a cross-lagged model [41,42], each construct is specified to influence itself over time (“autoregressive effect”) and cross over to influence the other construct at a subsequent time (“cross-lagged effect”), with the variance/residual covariance of subsequent constructs measured at the same time set to covary. This type of model solves several issues of cross-sectional models [43]. Several fit indices were used to assess model fit: χ^2^ and its ratio with the degrees of freedom (χ^2^ /df), the Comparative Fit index (CFI), the Tucker Lewis Index (TLI) and the root mean square error of approximation (RMSEA). If χ^2^ is not significant, the model is adequate. A good fit of the model to the data is also indicated when the χ^2^/df (degrees of freedom) ratio is less than three [44]. For CFI and TLI, values above 0.95 are preferred, and values close to 0.90 are considered acceptable [45,46]. RMSEA values below 0.05 reveal a good fit, whereas values between 0.05 and 0.08 reveal an acceptable fit [47].

All analyses were performed with IBM SPSS Statistics Version 22.0 (IBM Corp, Armonk, NY, USA) [48] and Mplus 7.0 (Los Ángeles, CA, USA) [49].

## 3. Results

### 3.1. Descriptive Statistics and Bivariate Correlations

Table 1 shows the descriptive statistics (mean and standard deviations) and correlations between the study variables at each of the three measurement times. As the table shows, while mean values for EK and ER increased over time, mean values for L/N decreased. Wilcoxon matched pairs tests were conducted to determine if these differences between means were significant across time. Results showed that mean differences from T1 to T3 were statistically significant for all three variables: EK (*Z_T1-T2_* = −4.38, *p* < 0.001, *r* = 0.045; *Z_T1-T3_* = −7.66, *p* < 0.001, *r* = 0.80; *t_T2-T3_* = −6.23, *p* < 0.001, *r* = 0.63), ER (*Z_T1-T2_* = −4.04, *p* < 0.001, *r = 0*.42; *Z_T1-T3_* = −6.98; *p* < 0.001, *r* = 74; *Z_T2-T3_* = −5.86, *p* < 0.001, *r* = 0.59) and L/N (*Z_T1-T2_* = 6.58, *p* < 0.001, *r = 0*.68; *Z_T1-T3_* = 6.34, *p* < 0.001, *r* = 0.35; *Z_T2-T3_* = 3.50, *p* < 0.001, *r* = 0.035).

Regarding the bivariate correlations, ER at T1 was positively associated with EK at T2, and ER was also associated with EK at T3. In contrast, L/N at T1 correlated negatively to ER at T3. The largest correlations were found among the same variables across time, specifically, between ER at T2 and ER at both T1 and T3, and between L/N at T2 and L/N at both T1 and T3, with all Pearson correlations values higher than 0.40.

### 3.2. Cross-Lagged Model of Emotion Knowledge and Emotion Regulation

**Figure 1:**
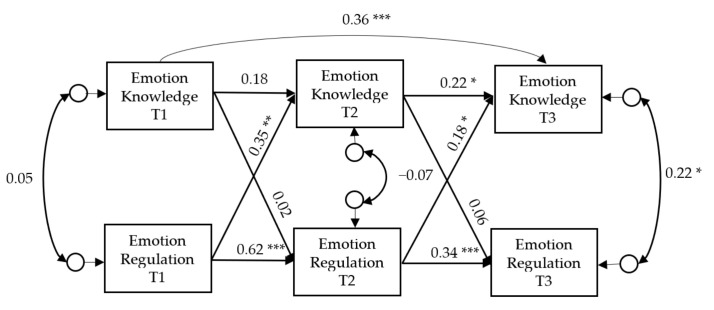
Cross-lagged model for testing the longitudinal association between emotion knowledge and emotion regulation across the three measurement times. Values are standardized coefficients. * *p* < 0.05; ** *p* < 0.01; *** *p* < 0.001.

The cross-lagged model that estimated the associations between EK and ER across time, after controlling for their stability over time [41], showed a poor fit for the data: χ^2^ (4) = 19.024, *p* < 0.001; the χ^2^/df ratio = 4.76, CFI = 0.844; TLI = 0.453; and RMSEA = 0.186, 90% CI [0.117–0.273]. A review of the modification indices (MIs) revealed an autorregresive path for EK from T1 to T3 that showed the largest value. Thus, a second cross-lagged model, shown in Figure 1, was constructed where this parameter was added. This new model yielded an adequate fit for the data: the χ^2^ value obtained was not significant (χ^2^ (3) = 4.665, *p* = 0.198), the χ^2^/df ratio = 1.56 (below 3), CFI was 0.983 and TLI was 0.919, which were above 0.95 and 0.90, respectively. In addition, the RMSEA did not exceeded the cut-off value, its value being .071 90% CI [0.000–0.190].

As Figure 1 shows, the autoregressive effects for EK were significant between T1 and T3, and between T2 and T3. For ER, all the autoregressive paths were significant, showing that this construct was stable over time.

Two cross-lagged effects from ER (T1) to EK (T2) and from ER (T2) to EK (T3) were statistically significant (0.35, *p* < 0.01, and 0.18, *p* < 0.05, respectively). Neither of the two cross-lagged effects from EK to ER were statistically significant.

This model explained 15.9% (*p* < 0.05) and 26.3% (*p* < 0.01) of the variance in EK at T2 and T3, respectively.

### 3.3. Cross-Lagged Model of Emotion Knowledge and Emotion Lability/Negativity

**Figure 2:**
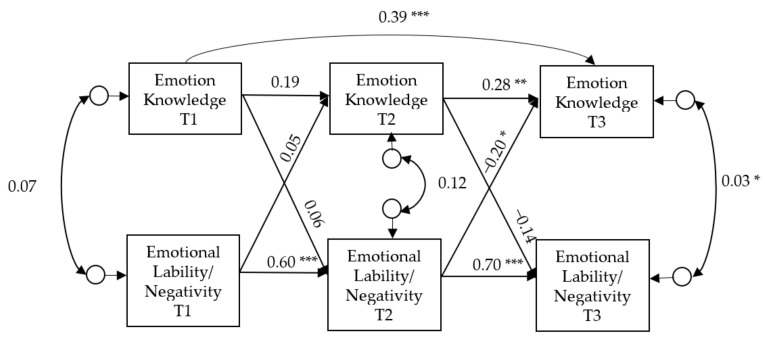
Cross-lagged model for testing the longitudinal association between emotion knowledge and emotion lability/negativity across the three measurement times. Values are standardized coefficients. * *p* < 0.05; ** *p* < 0.01; *** *p* < 0.001.

The associations between EK and L/N over time, like those between EK and ER, also showed a poor fit for the data in the cross-lagged model: χ^2^ (4) = 18.525, *p* < 0.05, the χ^2^/df ratio = 4.63, CFI = 0.891 and TLI = 0.618, RMSEA = 0.183 90% CI [0.104–0.270]. Again, the review of the modification indices (MIs) revealed that the autorregresive path for EK from T1 to T3 showed the highest value. Consequently, a second cross-lagged model was constructed where this parameter was added. This model is presented in Figure 2. The fit indices of this new model showed an excellent fit to the data, specifically: the χ^2^ value obtained was not significant (χ^2^ (3) = 3.751, *p* = 0.290), the χ^2^/df ratio = 1.25 (below 3), CFI was 0.994 and TLI was 0.974, both values above 0.95 and RMSEA was 0.048 90% CI [0.000–0.175], below the standard criteria.

As Figure 2 shows, the autoregressive effects for EK were significant between T1 and T3, and between T2 and T3. All the autoregressive paths were significant for L/N, showing that this construct, like the ER construct, was temporally stable.

Only one cross-lagged effect from L/N (T2) to EK (T3) was statistically significant (−0.20, *p* < 0.05). There were no significant cross-lagged effects from EK to L/N.

This model explained 3.8% (*p* = 0.326) and 27.8% (*p* < 0.01) of the variance in EK at T2 and T3, respectively. Additionally, it explained 36.6% (*p* < 0.001) and 48% (*p* < 0.05) of the variance in L/N at T2 at T3, respectively.

## 4. Discussion

In this study we explore the longitudinal relationships between two closely related skills, emotion regulation and emotion knowledge. In the measure used, emotion regulation is further divided into two factors, emotion regulation and lability/negativity. Dividing ER into two factors allows us to make a more detailed analysis of this skill in relation to emotion knowledge. The results show that ER is a predictor of EK and L/N correlates negatively with EK. In terms of the measurement times, we observed a predictive relationship between ER at age 3 and EK at age 4, and between ER at age 4 and EK at age 5. Regarding L/N, a negative correlation is observed between L/N at age 3 and EK one year later, and between L/N at age 4 and EK one year later. In other words, the results suggest that ER is a skill that precedes and predicts EK during preschool years.

Our results contrast with those reported in other studies. Denham et al. [33] found a predictive relationship between attention control and EK, that is, cool executive functions. However, they found no correlation between hot executive functions and EK. Other studies, meanwhile, have found inverse relationships between the two variables, with EK being the precursor and predictor of ER [3,29]. These apparently conflicting results could be explained by the type of study and the time interval between measurements. We are not aware of any previous studies that include three-year longitudinal measurements with long time lags. We believe that measurements taken over three years could present a clearer picture of the role that ER plays in other variables linked to emotional competence, such as EK.

From a theoretical point of view, some authors interpret the role of ER as one of the first, basic skills of emotional competence. Thompson [50], for example, in a study of self-regulation in early childhood, calls emotion a dynamic of the child’s responses. This emotional dynamic has characteristics that are related to the baby’s temperament (i.e., intensity of emotion, emotional lability, latency, persistence of emotional response, etc.). Furthermore, according to Saarni [51], the baby’s *dynamic* or emotional reactions influence the emotional responses of the adult (i.e., from indifference to strong empathy that compels them to reach out to the child). The emotional response of the adult is one of the variables with the greatest influence on the emotion regulation of the child. According to attachment theorists, the adult response to the baby’s emotional dynamics generates the first framework of emotion regulation [52]. Thus, babies with secure relational frameworks regulate their emotions in one way and babies with insecure frameworks do so in a very different way [53,54]. These relational frameworks even determine the display of emotion regulation skills in adulthood [55]. This suggests that when we talk about self-regulation in early childhood, without underestimating the attentional aspects involved, we are basically referring to emotion regulation that coexists over time with the first emotion recognition skills attributed to babies.

From other perspectives, emotional expressions are taken to be (non-linguistic) signs that facilitate communication and regulation of behaviour before the first words appear [56,57]. Before and during language acquisition, emotion and facial/body expression are the first signs. As studies in preverbal communication show, gestures become the indicators that the child follows and uses in different situations [58]. Far from disappearing with language acquisition, these indicators accompany or even replace these skills [59]. In addition, certain emotional displays that we exhibit during communicative exchanges contribute to other people’s perception of us. Without this emotional expression, the image is incomplete. In this way, the expression of emotions become social signs [60].

These social signs, termed interpersonal regulators by Holodynski [56], are extremely useful during child development. Classic studies, such as the visual abyss [61], have already shown that the adult’s facial expressions guide the actions of the child, and likewise, children learn to use their emotional expressions as signs to satisfy their demands or ensure their well-being. In these preliminary interactions or “affective encounters”, as Reddy calls them [62], adults draw on their own cultural conventions to shape the child’s expressions into optimal responses for social relations in their specific cultural context. Affective encounters give the child emotion regulation strategies. The child appropriates these strategies and uses them autonomously in their individual and social daily life. Thus, long before they understand the role that mental states play in emotions, for example, children have already come a long way towards achieving emotion regulation. These two variables must be reciprocal, so that one influences the other and vice versa. However, our results can be interpreted from the perspective of some approaches to early development, and suggest that ER may be the first basic building block of EK.

### Weaknesses and Strengths of the Study

One of the weaknesses of this study is that ER is measured on the basis of a context-specific evaluation carried out by the children’s teachers, and the regulatory skills exhibited by the child in the school context might differ from those displayed in the family context [63,64]. Nevertheless, earlier studies also indicate that teachers are more perceptive evaluators of the subtle changes that occur in children [65]. On the other hand, we believe that the school context could be more demanding in terms of emotion regulation than the family context, and therefore teachers can probably give a more balanced assessment of the real capacities of the children. Another limitation is the sample, which is non-probabilistic and limits external validity.

The strength of this study is its longitudinal design. Some authors claim that this methodology is a prerequisite to understanding the developmental trajectory of emotion competence [66]. Very few longitudinal studies have been published; however, they more clearly reflect the changes that occur in the development of skills and how these relate to other variables over time. An understanding of how and when the correlations between variables occur can help design more effective intervention programs.

## 5. Conclusions

Our objective is to explore the relationship between ER and EK in preschoolers by conducting a longitudinal study using 3 time measurements in a sample of children aged between 3 and 6 years. The results show that ER at 3 years predicts EK at 4 and 5 years. In other words, children with high ER scores at 3 years had a better EK at 4 and 5 years. In contrast, children high in L/N at 3 years had worse EK results at 4 and 5 years. These results show the importance of ER in the first years of life and particularly in the preschool years. This is the time when children build their socio-emotional skills and their executive functions. ER is part of both of these competencies. These results contribute to the understanding of socio-emotional development and, specifically, to the relationship between main variables included in this field. Knowing how and at what point in developmental time ER correlates with other variables can contribute to the design of effective educational programs that strengthen this important skill. In this sense, educational programs for 2 and 3-year-old children could emphasize deep training in regulation abilities. Improving this skill during the first years of life in order to assess its effect on other social and academic skills or on emotional knowledge would be an efficient way of sustaining these results. In addition, these findings may be useful for daily clinical practice with patients and families (e.g., by creating targeted interventions according to different ages).

## Figures and Tables

**Table 1:** Descriptive statistics and correlations among the study variables.

Variables		T1 (*n* = 97)			T2 (*n* = 102)			T3 (*n* = 102)		
		EK	ER	L/N	EK	ER	L/N	EK	ER	L/N
T1	EK	---								
	ER	0.02	---							
	L/N	0.05	0.04	---						
T2	EK	0.19	0.35 **	0.08	---					
	ER	0.07	0.61 **	0.07	0.17	---				
	L/N	−0.03	0.06	0.61 **	0.13	0.06	---			
T3	EK	0.40 **	0.12	−0.21	0.31 **	−0.01	−0.14	---		
	ER	0.00	0.36 **	−0.22 *	0.12	0.56 **	−0.17	0.28 **	---	
	L/N	−0.04	−0.06	0.38 **	−0.04	0.06	0.69 **	−0.16	−0.18	---
Mean		3.81	26.36	26.81	4.69	27.60	22.59	5.97	29.75	21.01
SD		1.38	2.92	6.15	1.25	3.01	5.02	1.36	2.47	5.27

Note: T1 = Time 1; T2 = Time 2; T3 = Time 3. EK = emotion knowledge; ER = emotion regulation; L/N = emotion lability/negativity. * *p* < 0.05; ** *p* < 0.001.

## References

[1] Denham S.A. (1998). Emotional Development in Young Children.

[2] Denham S.A. (2010). Emotion regulation: Now you see it, now you don’t. Emot. Rev..

[3] Izard C., Stark K., Trentacosta C., Schultz D. (2008). Beyond Emotion Regulation: Emotion Utilization and Adaptive Functioning. Child Dev. Perspect..

[4] Jones D.E., Greenberg M., Crowley M. (2015). Early Social-Emotional Functioning and Public Health: The Relationship Between Kindergarten Social Competence and Future Wellness. Am. J. Public Health.

[5] Sánchez-Puerta M.L., Valerio A., Gutiérrez Bernal M. (2016). Taking Stock of Programs to Develop Socioemotional Skills: A Systematic Review of Program Evidence.

[6] Taylor R.D., Oberle E., Durlak J.A., Weissberg R.P. (2017). Promoting Positive Youth Development Through School-Based Social and Emotional Learning Interventions: A Meta-Analysis of Follow-Up Effects. Child Dev..

[7] Cicchetti D., Ackerman B.P., Izard C.E. (1995). Emotions and emotion regulation in developmental psychopathology. Dev. Psychopathol..

[8] Durlak J.A., Weissberg R.P., Dymnicki A.B., Taylor R.D., Schellinger K.B. (2011). The impact of enhancing students’ social and emotional learning: A meta-analysis of school-based universal interventions. Child Dev..

[9] Trentacosta C.J., Fine S.E. (2010). Emotion Knowledge, Social Competence, and Behavior Problems in Childhood and Adolescence: A Meta-Analytic Review. Soc. Dev. Oxf. Engl..

[10] Denham S.A., Blair K.A., DeMulder E., Levitas J., Sawyer K., Auerbach-Major S., Queenan P. (2003). Preschool emotional competence: Pathway to social competence?. Child Dev..

[11] Garner P.W., Waajid B. (2012). Emotion Knowledge and Self-Regulation as Predictors of Preschoolers’ Cognitive Ability, Classroom Behavior, and Social Competence. J. Psychoeduc. Assess..

[12] Giménez-Dasí M., Fernández-Sánchez M., Quintanilla L. (2015). Improving social competence through emotion knowledge in 2-year-old children: A pilot study. Early Educ. Dev..

[13] Caprara G.V., Barbaranelli C., Pastorelli C., Bandura A., Zimbardo P.G. (2000). Prosocial foundations of children’s academic achievement. Psychol. Sci..

[14] Compas B.E., Jaser S.S., Bettis A.H., Watson K.H., Gruhn M.A., Dunbar J.P., Williams E., Thigpen J.C. (2017). Coping, emotion regulation, and psychopathology in childhood and adolescence: A meta-analysis and narrative review. Psychol. Bull..

[15] Robson D.A., Allen M.S., Howard S.J. (2020). Self-regulation in childhood as a predictor of future outcomes: A meta-analytic review. Psychol. Bull..

[16] Smithers L.G., Sawyer A.C.P., Chittleborough C.R., Davies N.M., Davey Smith G., Lynch J.W. (2018). A systematic review and meta-analysis of effects of early life non-cognitive skills on academic, psychosocial, cognitive and health outcomes. Nat. Hum. Behav..

[17] Berking M., Wupperman P. (2012). Emotion regulation and mental health: Recent findings, current challenges, and future directions. Curr. Opin. Psychiatry.

[18] Eisenberg N., Cumberland A., Spinrad T.L., Fabes R.A., Shepard S.A., Reiser M., Murphy B.C., Losoya S.H., Guthrie I.K. (2001). The relations of regulation and emotionality to children’s externalizing and internalizing problem behavior. Child Dev..

[19] Kim J., Cicchetti D. (2010). Longitudinal pathways linking child maltreatment, emotion regulation, peer relations, and psychopathology. J. Child Psychol. Psychiatry.

[20] Lonigan C.J., Spiegel J.A., Goodrich J.M., Morris B.M., Osborne C.M., Lerner M.D., Phillips B.M. (2017). Does Preschool Self-Regulation Predict Later Behavior Problems in General or Specific Problem Behaviors?. J. Abnorm. Child Psychol..

[21] Pons F., Harris P.L., de Rosnay M. (2004). Emotion comprehension between 3 and 11 years: Developmental periods and hierarchical organization. Eur. J. Dev. Psychol..

[22] Giménez-Dasí M., Quintanilla L., Lucas-Molina B. (2018). Scripts or components? A comparative study of basic emotion knowledge in Roma and non-Roma children. Early Educ. Dev..

[23] Hoehl S., Lagattuta K.H. (2013). Emotion Processing in Infancy. Children and Emotion. New Insights into Developmental Affective Sciences.

[24] Widen S.C., Barrett L.F., Lewis M., Haviland J.M. (2018). The Development of Children’s Concepts of Emotion. Handbook of Emotions.

[25] Thompson R.A., Calkins S.D., Bell M.A. (2010). Feeling and understanding through the prism of relationships. Child Development at the Intersection of Emotion and Cognition.

[26] Pons F., Lawson J., Harris P.L., Rosnay M.D. (2003). Individual differences in children’s emotion understanding: Effects of age and language. Scand. J. Psychol..

[27] Pine D.S., Fox N.A. (2015). Childhood antecedents and risk for adult mental disorders. Annu. Rev. Psychol..

[28] Morris A.S., Silk J.S., Steinberg L., Myers S.S., Robinson L.R. (2007). The Role of the Family Context in the Development of Emotion Regulation. Soc. Dev. Oxf. Engl..

[29] Di Maggio R., Zappulla C., Pace U. (2016). The relationship between emotion knowledge, emotion regulation and adjustment in preschoolers: A mediation model. J. Child Fam. Stud..

[30] Denham S.A., Bassett H.H., Way E., Mincic M., Zinsser K., Graling K. (2012). Preschoolers’ emotion knowledge: Self-regulatory foundations, and predictions of early school success. Cognit. Emot..

[31] Schultz D., Izard C.E., Ackerman B.P., Youngstrom E.A. (2001). Emotion knowledge in economically disadvantaged children: Self-regulatory antecedents and relations to social difficulties and withdrawal. Dev. Psychopathol..

[32] Willoughby M., Kupersmidt J., Voegler-Lee M., Bryant D. (2001). Contributions of hot and cool self-regulation to preschool disruptive behavior and academic achievement. Dev. Neuropsychol..

[33] Denham S.A., Bassett H.H., Zinsser K. (2012). Early childhood teachers as socializers of young children’s emotional competence. Early Child. Educ. J..

[34] Johnson M.H., Jones E.J.H., Gliga T. (2015). Brain adaptation and alternative developmental trajectories. Dev. Psychopathol..

[35] Leach P., Leach P. (2018). Transforming Infant Wellbeing: Research, Policy and Practice For the First 1001 Critical Days.

[36] Shonkoff J.P. (2017). Rethinking the Definition of Evidence-Based Interventions to Promote Early Childhood Development. Pediatrics.

[37] Pons F., Harris P.L. (2000). Test of Emotion Comprehesion.

[38] Pons F., Harris P. (2005). Longitudinal change and longitudinal stability of individual differences in children’s emotion understanding. Cognit. Emot..

[39] Shields A., Cicchetti D. (1997). Emotion regulation among school-age children: The development and validation of a new criterion Q-sort scale. Dev. Psychol..

[40] Tomczak M., Tomczak E. (2014). The need to report effect size estimates revisited. An overview of some recommended measures of effect size. Trends Sport Sci..

[41] Cole D.A., Maxwell S.E. (2003). Testing Mediational Models With Longitudinal Data: Questions and Tips in the Use of Structural Equation Modeling. J. Abnorm. Psychol..

[42] Liu Y., Mo S., Song Y., Wang M. (2016). Longitudinal analysis in occupational health psychology: A review and tutorial of three longitudinal modeling techniques. Appl. Psychol. Int. Rev..

[43] Maxwell S.E., Cole D.A. (2007). Bias in cross-sectional analyses of longitudinal mediation. Psychol. Methods.

[44] Kline R.B. (USA 2005). Principles and Practice of Structural Equation Modeling.

[45] Bentler P.M. (1990). Comparative fit indexes in structural models. Psychol. Bull..

[46] Kline R.B. (2016). Methodology in the social sciences. Principles and Practice of Structural Equation Modeling.

[47] Browne M.W., Cudeck R. (1992). Alternative Ways of Assessing Model Fit. Sociol. Methods Res..

[48] IBM Corp (2013). IBM SPSS Statistics for Windows.

[49] Muthén L.K., Muthén B.O. (2012). MPLUS.

[50] Thompson R.A. (1991). Emotional regulation and emotional development. Educ. Psychol. Rev..

[51] Saarni C. (1999). The Development of Emotional Competence.

[52] Mikulincer M., Shaver P.R., Corr P.J., Matthews G. (2009). Attachment theory: II Developmental, psychodynamic and optimal-functioning aspects. The Cambridge Handbook of Personality Psychology.

[53] Diener M.L., Mangelsdorf S.C., McHale J.L., Frosch C.A. (2002). Infants’ Behavioral Strategies for Emotion Regulation With Fathers and Mothers: Associations With Emotional Expressions and Attachment Quality. Infancy.

[54] Cooke J.E., Kochendorfer L.B., Stuart-Parrigon K.L., Koehn A.J., Kerns K.A. (2019). Parent–child attachment and children’s experience and regulation of emotion: A meta-analytic review. Emotion.

[55] Girme Y.U., Jones R.E., Fleck C., Simpson J.A., Overall N.C. (2020). Infants’ attachment insecurity predicts attachment-relevant emotion regulation strategies in adulthood. Emotion.

[56] Holodynski M. (2013). The internalization theory of emotions: A cultural historical approach to the development of emotions. Mind Cult. Act..

[57] Holodynski M., Seeger D. (2019). Expressions as signs and their significance for emotional development. Dev. Psychol..

[58] Butterworth G., Kita S. (2003). Pointing is the royal road to language for babies. Pointing: Where Language, Culture, and Cognition Meet.

[59] Goldin-Meadow S. (1999). The role of gesture in communication and thinking. Trends Cognit. Sci..

[60] Hareli S., Hess U. (2012). The social signal value of emotions. Cognit. Emot..

[61] Sorce J.F., Emde R.N., Campos J.J., Klinnert M.D. (1985). Maternal emotional signaling: Its effect on the visual cliff behavior of 1-year-olds. Dev. Psychol..

[62] Reddy V. (2019). Meeting infant affect. Dev. Psychol..

[63] Bernie A., Carlson S.M., Whipple N. (2010). From External Regulation to Self-Regulation: Early Parenting Precursors of Young Children’s Executive Functioning. Child Dev..

[64] Graziano P.A., Reavis R.D., Keane S.P., Calkins S.D. (2007). The role of emotion regulation in children’s early academic success. J. Sch. Psychol..

[65] Sarmento-Henrique R., Lucas-Molina B., Quintanilla-Cobián L., Giménez-Dasí M. (2017). La evaluación multi-informante de la regulación emocional en edad preescolar: Un estudio longitudinal. Psicol. Educ..

[66] Thompson R.A., Goodvin R., Brownell C.A., Kopp C.B. (2007). Taming the tempest in the teapot: Emotion regulation in toddlers. Socioemotional Development in the Toddler Years: Transitions and Transformations.

